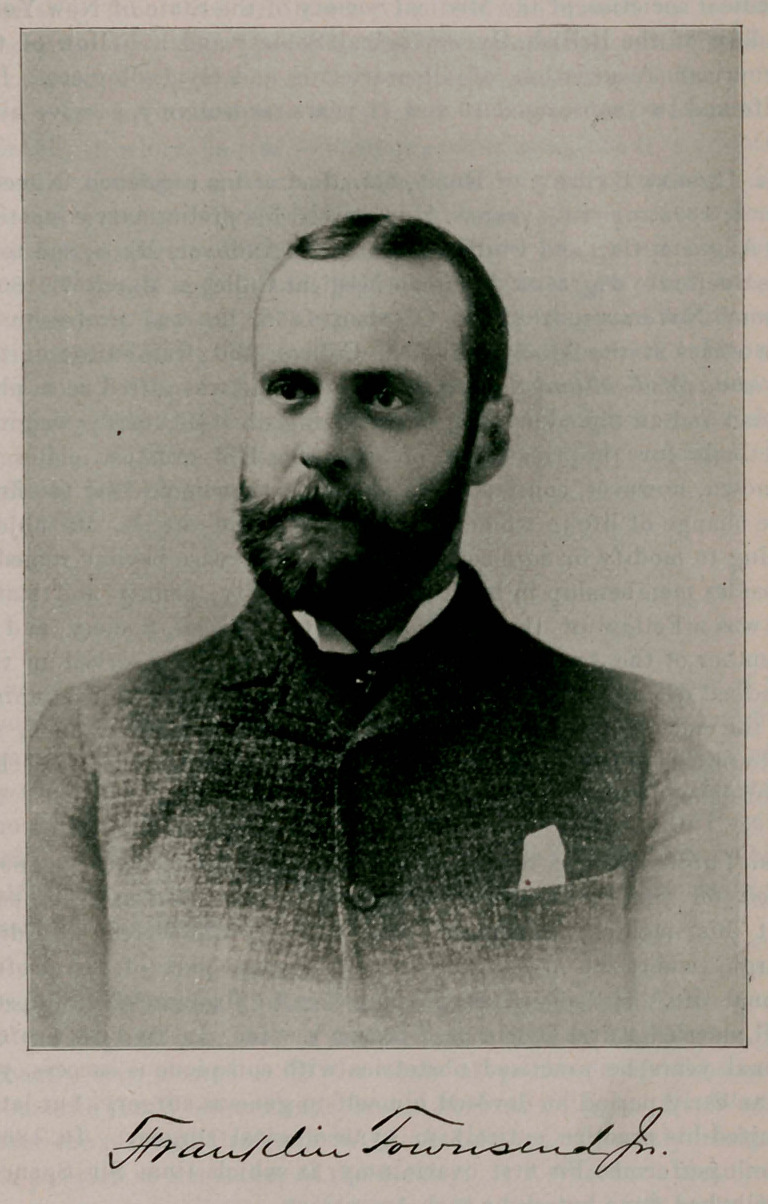# Dr. Franklin Townsend, Jr.

**Published:** 1895-12

**Authors:** 


Dr. Franklin Townsend, Jr., died at his residence, No. 2 Park Place, Albany, October 31, 1895, aged 41 years. He received his early education in Albany, took a full academic course at Williams' College, graduating with the degree of A. B. in 1873, and received his doctorate degree from the College of Physicians and Surgeons in New York in 1876. He spent a year in Europe soon afterward, where he pursued his medical studies and on his return began the practice of his profession in his native city, Albany. In 1880, he was appointed lecturer on physiology in Albany Medical College and the next year was elected to the full professorship in that chair, which he held until compelled to relinquish it through failing health. He was distinguished as a teacher, respected by his pupils and loved by his colleagues. His public and private patients were strongly attached to him, to whose interests he always unselfishly devoted his conspicuously bright and skilful talents.

In 1877, Dr. Townsend married Margaret W. Reynolds, daughter of John H. Reynolds, of Albany, a talented member of the bar and a conspicuous ornament to the bench.

Dr. Townsend possessed a most amiable disposition and his home-life was something delightful to behold. Whoever has partaken of the hospitality that was so lavishly and elegantly dispensed at No. 2 Park Place will not soon forget the beautiful picture that Dr. and Mrs. Townsend presented in the lights and shades of a cultured home-circle.

Dr. Townsend's contributions to the literature of medicine always exhibited a high order of professional knowledge and great research. He wrote easily and well, but never effusively. It was not easy to obtain his consent to write, but when finally he gave it, his pen moved to some purpose. One of his strongest papers is on the pathology of extrauterine pregnancy, published in Volume I., Transactions of American Association of Obstetricians and Gynecologists, 1888. One of his last professional papers was a memorial of Arthur Wellesley Edis, published in the Transactions of the above mentioned association, Volume VII., 1894. It was a touching tribute of love from one friend to the memory of another, couched in simple but elegant English, and some of the sentences might well replace these that are now writing for and of the man who wrote the other.

At the time of his death, Dr. Townsend was emeritus professor of physiology in Albany Medical College; obstetrician to the

Franklin Townsend Jr.

Albany City hospital ; visiting obstetrician and gynecologist to St. Peter's hospital and visiting physician to the Albany Protestant orphan asylum. He was a member of the local city and county medical societies, of the Medical Society of the State of New York, Fellow of the British Gynecological Society and a Fellow of the American Association of Obstetricians and Gynecologists. His wife and two sons, aged 16 and 11 years respectively, survive him.

## Figures and Tables

**Figure f1:**